# A Rare Concurrence of Myelodysplastic Neoplasia and Tetrasomy 8 in a 3-Year-Old Bahraini Male

**DOI:** 10.7759/cureus.41988

**Published:** 2023-07-17

**Authors:** Zainab A Toorani, Ameera A Radhi, Merna M Hassan, Ameera A Aloraibi

**Affiliations:** 1 Pathology, Salmaniya Medical Complex, Manama, BHR; 2 Hematopathology, Salmaniya Medical Complex, Manama, BHR; 3 Medicine, Royal College of Surgeons in Ireland, Muharraq, BHR; 4 Pediatric Hematology/Oncology, Salmaniya Medical Complex, Manama, BHR

**Keywords:** myelodysplastic neoplasia, peripheral blood smear, bone marrow biopsy, cytopenia, tetrasomy 8, acute myeloid leukemia

## Abstract

Myelodysplastic neoplasia (MDS) is a group of stem cell disorders involving ineffective hematopoiesis. It can be associated with an increased risk of progression toward acute myeloid leukemia (AML). In Bahrain, MDS is the fifth most common primary hematologic malignancy. MDS has an annual incidence of up to 4 million cases. Some of the presenting signs and symptoms of MDS are often nonspecific, such as fatigue, pallor, malaise, fevers, bleeding, bruising, weight loss, and anorexia. Approximately 40% of patients with MDS progress to AML. This paper outlines a case of a 3-year-old Bahraini male (known to have sickle cell trait) who presented to the emergency department of Salmaniya Medical Complex with a five-day history of fever, congested throat, left ear pain, and abdominal pain. He had one episode of vomiting gastric content the previous day. He had previously gone to a private clinic with similar symptoms. Physical examination revealed a short neck and short stature, which was found to be below the 5^th^ percentile. He had generalized pallor and hepatosplenomegaly. A blood smear showed leukopenia and normochromic normocytic anemia. There were excessive blasts found which consisted of 17% of nucleated cells and few granulopoietic cells. Erythropoiesis was active with a few showing mild megaloblastic changes. There were rare megakaryocytes noted. Moreover, the bone marrow aspirate showed two populations on dim CD45. The first population consisted of 3.15% on dim CD45 comprising of hematogones which brightly expressed CD19, HLA-DR, CD79a, and dim CD10. The second population consisted of 14.85% on dim CD45 which expressed CD34, CD13, CD117, HLA-DR, and dim CD7. Based on the peripheral blood smear and bone marrow immunophenotyping findings, a diagnosis of myelodysplastic syndrome with excessive blasts was made, which soon transformed into a diagnosis of AML. Furthermore, increased levels of dysplastic changes and percentage of blasts in the peripheral blood smear and bone marrow lead to a higher possibility of transformation into AML. As per the WHO classification, a diagnosis of MDS needs evaluation of the morphology of blood and bone marrow.

## Introduction

Myelodysplastic neoplasia (MDS) is a group of stem cell disorders involving ineffective hematopoiesis. MDS presents with cytopenia or dysplasia in one or more of the major myeloid lineages [1]. It can be associated with inherited bone marrow failure syndromes and has an increased risk of progression to acute myeloid leukemia (AML) [2].

In Bahrain, MDS is the fifth most common primary hematologic malignancy [3]. Worldwide, MDS accounts for less than 5% of childhood hematologic malignancies, with an annual incidence of up to 4 million cases [4]. Although the genetic changes which predispose children to MDS are not known, the most common cytogenetic abnormality association is monosomy 7, present in about 30% of cases [2].

The presenting signs and symptoms of MDS are often nonspecific, with children presenting with signs of anemia and thrombocytopenia, such as fatigue, pallor, malaise, fevers, bleeding, bruising, weight loss, and anorexia [5]. Cases can also be discovered incidentally on routine laboratory testing or in the work up of suspected inherited bone marrow failure, with approximately 20% of cases being found this way [6].

Studies on the outcomes of childhood MDS are often limited due to a small sample size because of the rarity of the disease. Hematopoietic stem cell transplant (HSCT) is the only known curative therapy, and it is agreed by many that it has been the most effective way of improving survival [7,8].

## Case presentation

A 3-year-old Bahraini male, known to have sickle cell trait, presented to the emergency department with a five-day history of fever, congested throat, left ear pain, and abdominal pain. He had one episode of vomiting gastric content the previous day. He had previously gone to a private clinic and was given analgesics and an antibiotic. Following lab investigations in the clinic, a low hemoglobin of 6.8 g/dL (normal range: 12-14.5 g/dL) was noted, and the patient was sent to the emergency department in the main tertiary hospital.

The patient’s family denied any history of cough, shortness of breath, chest pain, or palpitations. There was no decreased feeding or activity, and no changes in bowel or urinary habits. There was no recent travel or sick contact history.

The patient is a product of full-term normal vaginal delivery, with no natal or antenatal complications. Vaccinations are up to date. There are no known allergies.

On physical examination, the patient was vitally stable, aside from a fever. He had a short neck and short stature, which is found to be below the 5th percentile. He had generalized pallor. There was hepatosplenomegaly which continued to increase with time. The liver was palpated to be 4 cm below the right costal margin, and the spleen was palpated 3 cm below the left costal margin. The tonsils were mildly congested without the presence of exudate or follicles; tympanic membranes were clear bilaterally. Cardiovascular examination was normal.

An abdominal ultrasound showed a liver span of 11.9 cm in long access with a homogenous echo pattern. The spleen was also enlarged, measuring 14 cm in long access with a homogenous echo pattern. No focal mass lesions were noted in either. There were no abdominal abnormalities visualized on ultrasound.

Some of the significant and important investigations that were done during the patient's initial presentation are outlined in Table 1.

**Table 1:** Laboratory investigations that were performed during the patient's initial presentation to the hospital

Laboratory investigations done on the 14^th^ of October 2022	Result	Normal reference range
Hemoglobin levels	6.7 g/dL	13.5-15.5 g/dL
Mean cell volume (MCV)	77.9 fL	82-97 fL
Mean cell Hemoglobin (MCH)	25.1 pg	27-33 pg
Mean cell Hemoglobin concentration (MCHC)	32.2 g/dL	30-37 g/dL
Platelets	252 x10^9/L	150-400 x10^9/L
Red blood cells count	2.66 x10^12/L	4.2-5.8 x10^12/L
White blood cells count	5.38 × 10^9/L	3.6-9.6 x 10^9/L
Manual differential of neutrophils	31%	40-60 %
Manual differential of lymphocytes	67%	20-40 %
Manual differential of monocytes	1%	2-8%
Manual differential of atypical lymphocytes	1%	<1 %

LDH continued to significantly increase over time, as demonstrated in Table 2.

**Table 2:** A summary of the lactate dehydrogenase levels

Laboratory investigation performed according to date	7^th^ November 2022	19^th^ November 2022	26^th^ December 2022	4^th^ January 2023	5^th^ January 2023
Lactate dehydrogenase (normal range: 100-300 U/L)	1104	1553	1860	2802	2877

Furthermore, other laboratory investigations such as ESR and CRP were not elevated. The iron levels were 45 µmol/L (normal range: 11.6-31.3 µmol/L) and ferritin levels were 136.3 µg/L (normal range: 16-323 µmol/L). 

On November 8, 2022, the peripheral blood smear showed a left shift with 4% blast cells along with nucleated red blood cells. The results for a bone marrow aspirate showed aparticulated diluted specimen along with few myeloid and erythroid cells, therefore a repeat of the procedure was recommended.

On November 20, 2022, a peripheral blood smear showed 4% blasts, and nucleated red blood cells were seen. Red blood cells showed microcytosis and hypochromia. Bilateral bone marrow aspirates showed aparticulated diluted specimens along with few lymphocytes and neutrophils. Therefore, the interpretation was inadequate for evaluation. Immunophenotyping was requested due to pancytopenia and a significant increase in LDH levels; it showed 3.8% of blasts on dim CD45 which expressed HLA-DR, CD117, and CD34. The possibility of MDS was noted, but the immunophenotyping did not support a diagnosis of leukemia.

On December 6, 2022, a bone marrow biopsy consisting of two specimens showed 98%-100% cellularity. The interstitium was infiltrated by immature cells consisting of nuclei with open chromatin. Some of these cells were arranged in clusters. There was normal hematopoiesis seen along with a marked increase in megakaryocytes. A repeat bone marrow aspirate study was advised for better evaluation and to rule out leukemia.

The chromosomal analysis, completed on December 8, 2022, indicated the existence of a pathological cell line with isolated tetrasomy 8, represented as 48,XY,+8,+8, according to the International System for Human Cytogenomic Nomenclature [9]. Unfortunately, further follow-up analysis was not done due to insufficient material for FISH analysis.

Bone marrow differentials on the December 28, 2022 consisted of 17% blasts, 8% metamyelocytes, 7% band forms, 2% neutrophils, 35% lymphocytes, and 30% normal blasts. The peripheral blood smear interpretation showed leukopenia with 10% blasts and normocytic anemia. It contained a single hypercellular bone marrow particle. The trails were moderately cellular showing blast cells accounting for 17% of all nucleated cells. Few granulopoietic cells were noted. Erythropoiesis was active, with some showing mild megaloblastic changes. Rare megakaryocytes were seen. Iron stain showed a single particle consistent with grade 2. Flow cytometry immunophenotyping of the bone marrow showed two populations on dim CD45. The abnormal population of cells consisted of MDS with excessive blasts and hematogones. The first population consisted of 3.15% on dim CD45 comprising of hematogones which brightly expressed CD19, HLA-DR, CD79a, and dim CD10. The second population consisted of 14.85% on dim CD45 which expressed CD34, CD13, CD117, HLA-DR, and dim CD7. Based on the peripheral blood smear, bone marrow immunophenotyping findings, and chromosomal analysis a diagnosis of MDS with excessive blasts was made. A diagnosis of AML was made shortly after. 

On December 29, 2022, the biopsy was repeated and showed 98% cellularity. The megakaryocytes were adequate in number, granulopoiesis was present, and erythropoiesis was seen. There was an increased number of immature cells noted in the interstitium. On immunohistochemical studies, the immature cells stained positively for CD34, CD117, and CD7. A diagnosis of myelodysplasia with excess blasts type 2 was reported.

On January 5, 2023, a peripheral blood smear showed 21% blasts. Flow cytometry immunophenotyping showed a population of abnormal cells on dim CD45 with 23.3% blasts which expressed HLA-DR, CD34, CD38, and dim CD13 and CD7. This was consistent with a diagnosis of leukemia. The patient was closely monitored and received weekly blood transfusions for consistently low hemoglobin. He was sent overseas where a course of chemotherapy followed by a bone marrow transplant was done. The patient is currently doing well.

## Discussion

MDS has been diagnosed in only 6% of those younger than fifty years of age, and diagnosed in approximately 90% of patients over the age of sixty years [5]. The most common subtype of myelodysplastic syndrome is refractory cytopenia in childhood (RCC). It accounts for nearly half of all MDS cases in children and adolescents [10]. The initial presentation includes symptoms related to pancytopenias [11,12], involving thrombocytopenia and neutropenia which is commonly seen in pediatric patients [6,10,13]. The patient described in this paper presented with anemia, fever, increased LDH levels, and hepatosplenomegaly. These are some of the resulting signs relatable to pancytopenia. Significant increases in LDH levels are used as a method of early disease progression, which in turn helps in risk stratification and options for interventional therapies [14].

The WHO classification for pediatric MDS diagnosis is used in around 89% of the North American Pediatric Aplastic Anemia Consortium (NAPAAC) centers. This classification comprises the dysplastic rate, uni- or multilineage dysplasia, presence of ring sideroblasts and its percentage, percentage of blast, and presence of any particular cytopenia [15]. 

The patient outlined in this paper falls under the MDS diagnosis category of refractory anemia with excess blasts (RAEB). This accounts for refractory anemia with excessive blasts, showing blood blasts of 10% and bone marrow blasts of 17% in the peripheral blood smear. This result was outlined after two aparticulated diluted samples. The bone marrow biopsy showed 98% cellularity consisting of megakaryocytes. Granulopoiesis and erythropoiesis were seen. There was an increased number of immature cells noted in the interstitium. The immature cells were positive on immunohistochemistry for CD34, CD117, and CD7.

**Figure 1:**
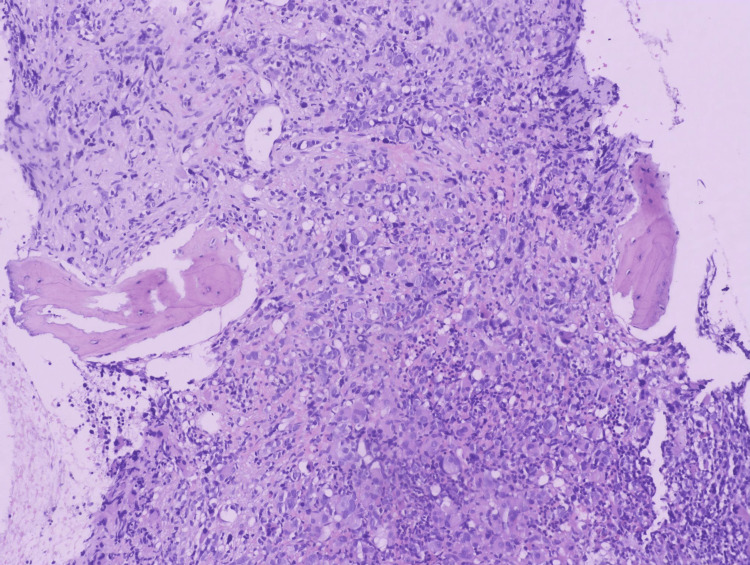
A bone marrow core biopsy showing increased cellularity consisting of hyperlobated and hypolobated megakaryocytes, micromegakaryocytes, and immature cells (H&E, Orig. x10)

**Figure 2:**
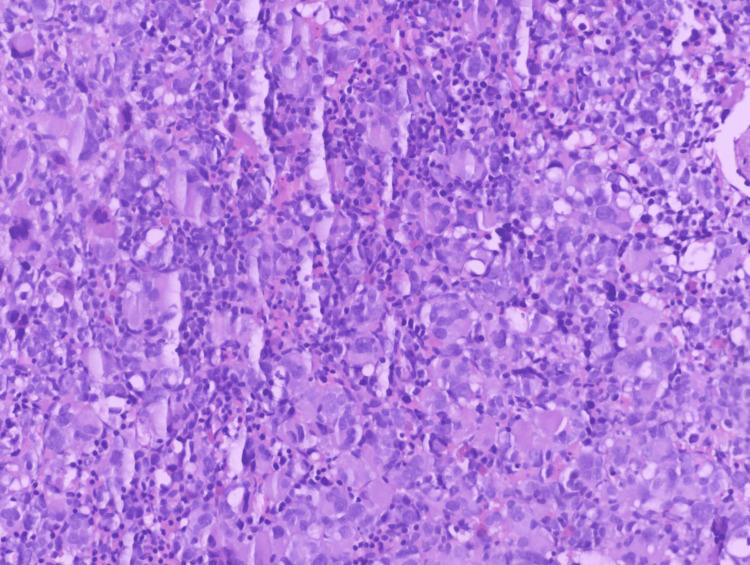
Higher power magnification of the bone marrow biopsy showing increased cellularity consisting of blasts and megakaryocytes (H&E, Orig. x20)

**Figure 3:**
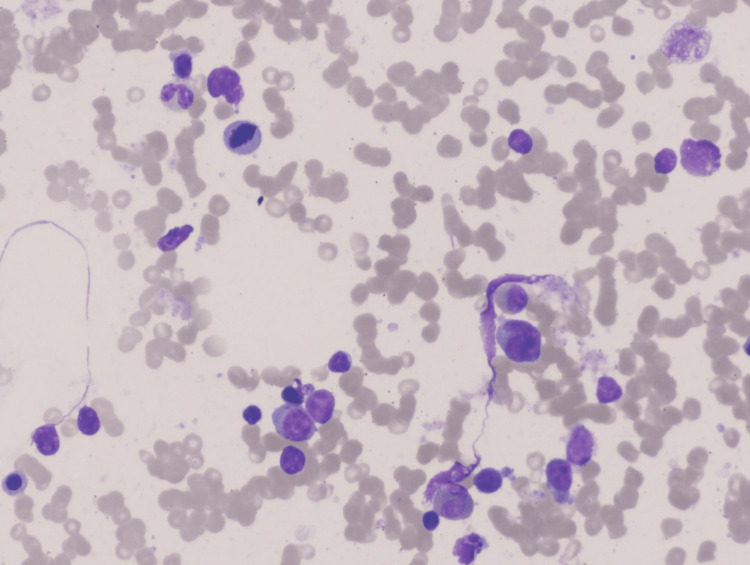
Bone marrow aspirate smear showing increased number of blasts (H&E, Orig. 40x)

**Figure 4:**
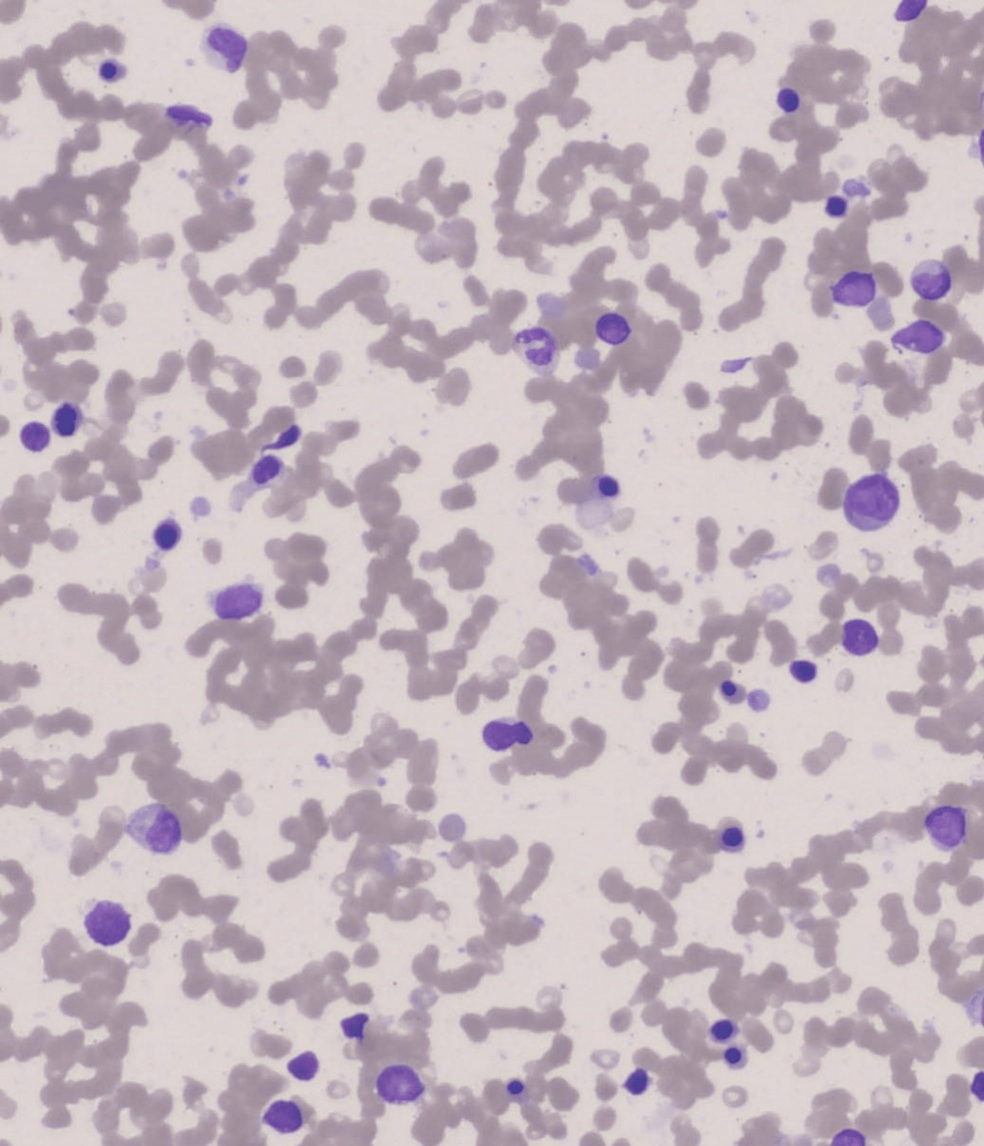
Bone marrow aspirate smear showing increased blasts along with rare agranular myeloid cells (H&E, Orig. 20x)

**Figure 5:**
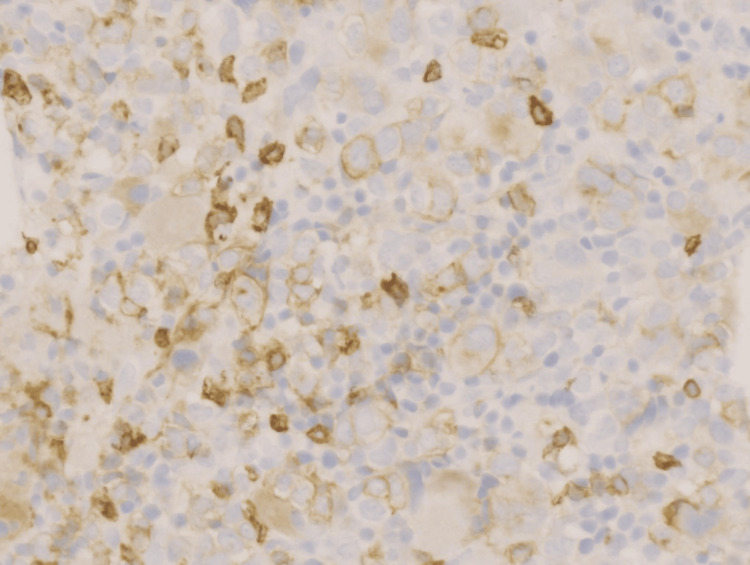
Blast cells showing immunostain positivity on CD7 (Orig. 40x)

**Figure 6:**
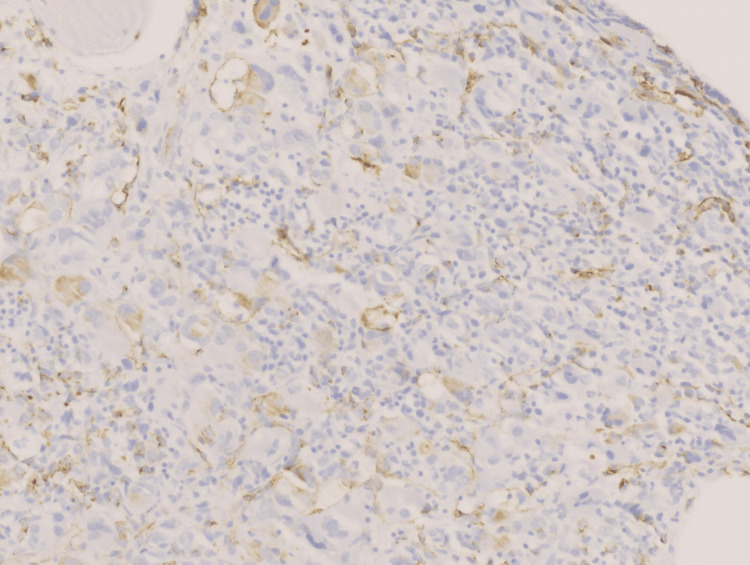
Blast cells showing immunostain positivity on CD34 (Orig. 40x)

**Figure 7:**
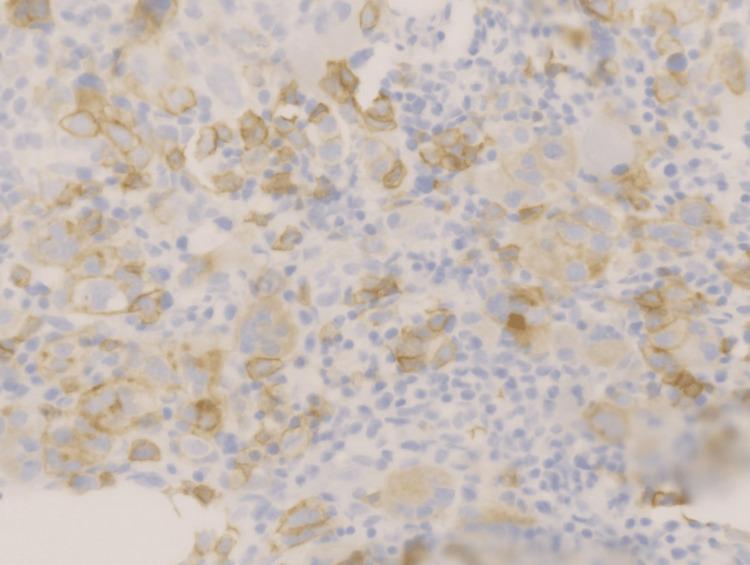
Blast cells showing immunostain positivity on CD117 (Orig. 40x)

Figures 1-7 demonstrate the patient's histological bone marrow biopsy, peripheral blood smear, and immunohistochemical studies.

Trisomy 8 is one of the most common chromosomal abnormalities correlated with hematological malignancies, particularly AML and MDS. The presence of tetrasomy 8 is a rare chromosomal abnormality which has also been associated with the latter [16,17]. Tetrasomy 8 has been linked to poor prognosis. This was previously evident in a case where the patient died after only six months of being diagnosed [16]. Most cases published in the literature, which are related to AML in childhood with a background of tetrasomy 8, have been diagnosed amongst the subtypes M0-M5 [16].

Studies have reported that there is a relationship between increased blast and leukocytic count in cases of tetrasomy 8 and trisomy 8 [17]. This association is demonstrated in our patient who showed an increased percentage of lymphocytes, band cells, blast cells, and atypical lymphocytes on presentation.

It is interesting to note that our patient is very young and carries a very rare karyotypic abnormality of 48, XY,+8,+8, which has not been previously reported in the literature. This resulted in an abnormal cytogenic result consisting of a pathological cell line with tetrasomy 8. In contrast with our case, only a handful of cases were published in the literature regarding tetrasomy 8, and most of the cases concerned older age group individuals. This paper, however, demonstrates a chromosomal abnormality identified in a 3-year-old Bahraini male which has not been previously reported. The abnormality was identified during the initial presentation of the patient in the form of physical signs, clinical signs, and laboratory results, which have been previously outlined. Unfortunately, the test that was carried out could not rule out the existence of other pathological cell lines. Additionally, the sample that was submitted was not enough for a further FISH test to be carried out. Shortly after being diagnosed with MDS, another flow cytometry immunophenotyping result showed a transformation to AML-MO. Approximately 40% of patients with MDS progress to AML [18]. There are many gene mutations involved that are responsible for the increased transformation of MDS to AML, including SRSF2 which is also responsible for decreased survival [18].

Moreover, there are many mutations found in MDS that involve both RNA splicing and epigenetic modification which includes ZRSR2, SF3B1, U2AF1, SRSF2, and DNAMT3A, ASXL1, and TET2 respectively [18].

MDS can demonstrate either numerical chromosome anomaly or structural abnormalities summing up to a total percentage of 30% to 50% in children. Amongst the 30% to 50%, numerical chromosomal abnormality accounted for the majority, and structural abnormalities were described in only 10% of the cases [6]. 

Treatment options initially involve assessing whether the patient is eligible for allogeneic stem cell transplantation, which is the most effective and curative method. Many other medical treatment options are subcategorized for lower-risk and higher-risk patients, including options like hypomethylating agents [10,19], erythropoiesis-stimulating agents, lenalidomide, immunosuppressive therapy, iron chelation, and intensive chemotherapy [10,6,19].

According to the latest WHO classification published in 2022, the term myelodysplastic syndrome has been renamed as MDS to outline a better clarity of the disease, although, the abbreviation of the disease remains the same [20]. MDS is defined by morphology and genetic abnormalities [20]. Therefore, there are many other novel targeted therapies against gene mutations like TP53, IDH1, and IDH2 [10,6,19].

## Conclusions

In conclusion, the concurrence of MDS and tetrasomy 8 in a 3-year-old is rare, especially when diagnosed in combination. During the patient’s initial presentation, it is important to note the precise clinical history, perform a thorough physical examination, and carry out significant blood tests which are key in determining further workup for a conclusive diagnosis. Moreover, many genetic factors play a role in the pathological cell lines in MDS and AML. Therefore, genetic analysis is important when MDS is diagnosed, and can further help in patient management. Our case report is written to widen perspectives and enhance a better understanding of this disease.
